# Pseudo-Peritoneal Carcinomatosis Presentation of a Crystal-Storing Histiocytosis With an Unmutated Monoclonal κ Light Chain

**DOI:** 10.1097/MD.0000000000001247

**Published:** 2015-08-14

**Authors:** Aude Aline-Fardin, Sebastien Bender, Bettina Fabiani, David Buob, Said Brahimi, Marie Christine Verpont, Mohamad Mothy, Pierre Ronco, Jean Jacques Boffa, Pierre Aucouturier, Laurent Garderet

**Affiliations:** From the AP-HP (AA-F, BF), Hôpital Saint Antoine, Department of Pathology, Paris; CNRS UMR 7276 Université de Limoges (SB, MCV), Hôpital Universitaire Dupuytren; AP-HP (DB), Hôpital Tenon, Department of Pathology, Paris; Université Pierre et Marie Curie-Paris 6 (DB), Paris; Centre hospitalier de Troyes (SB), Service D’Hématologie; AP-HP (MM), Hôpital Saint-Antoine, Service d’Hématologie Clinique et Thérapie Cellulaire, Paris; Université Pierre & Marie Curie (MM), Paris; INSERM (MM), UMRs, U938, Paris; AP-HP (PR, JJB), Hôpital Tenon, Department of Nephrology and Dialysis, Paris; Sorbonne Universités (PR, JJB), UPMC Univ Paris 06, UMR_S 1155; INSERM (PR, JJB), UMR_S 1155, Paris; INSERM (PA), UMRS 938, Hôpital Saint-Antoine, Paris; Université Pierre et Marie Curie-Paris6 (PA), Hôpital Saint-Antoine, Paris; INSERM (LG), UMR_S 938, Paris; AP-HP (LG), Hôpital Saint Antoine, Département d’hématologie et de thérapie cellulaire, Paris; and Université Pierre et Marie Curie-Paris6 (LG), Paris, France.

## Abstract

Crystal-storing histiocytosis (CSH) is a rare complication of monoclonal gammopathies caused by accumulation of crystalline material inside macrophages, and it may result in a variety of clinical manifestations depending on the involved organs. Although immunoglobulin κ light chains (LCs) seem to be the most frequent pathogenic component, very few molecular data are currently available.

A 69-year-old man presented with a very poor performance status. Remarkable features were mesenteric lymph node enlargement and proteinuria, including a monoclonal κ LC. Light and electron microscopy studies revealed the presence of crystals within macrophages in the lymph nodes, bone marrow, and kidney, leading to the diagnosis of CSH. The pathogenic κ LC variable domain sequence was identical to the germline Vk3-20^∗^01/Jk2^∗^01 gene segments, without any somatic mutation, suggesting an extra-follicular B cell proliferation.

The patient was successfully treated with 4 cycles of bortezomib and dexamethasone. After a 12-month follow-up, he remains in hematological and renal remission.

CSH may present as pseudo-peritoneal carcinomatosis and relate to a monoclonal κ LC encoded by an unmutated gene. Bortezomib-based therapy proved efficacious in this case.

## INTRODUCTION

Crystal-storing histocytosis (CSH) is a morphologically defined entity that features accumulation of crystals inside macrophages. These crystals are made up of a monoclonal immunoglobulin (Ig) light chain (LC), generally of κ type. CSH may involve either a single or multiple organs. It is usually associated with systemic manifestations and occasionally with renal involvement. Since the first description in 1978,^1^ >80 cases have been reported^2^; they were associated with B cell dyscrasias, mainly multiple myeloma, lymphoplasmacytic lymphoma, and, in more recent reports, with monoclonal gammopathy of undetermined significance (MGUS).^2^ In a few instances, CSH precedes the development of an overt lymphoproliferative disease.

The pathophysiology of monoclonal gammopathy-related CSH remains unclear.^3,4^ Very few molecular data are currently available concerning the LCs that seem responsible for macrophage activation and crystal storing.^5,6^

We report on a CSH case mimicking peritoneal carcinomatosis with severe loss of weight. The disease involved lymph nodes, bone marrow, and kidneys. A monoclonal κ LC was present in the urine, but a defined lymphoplasmacytic disease could not be demonstrated. The patient responded to a bortezomib-based therapeutic regimen.

## CASE REPORT

**FIGURE 1:**
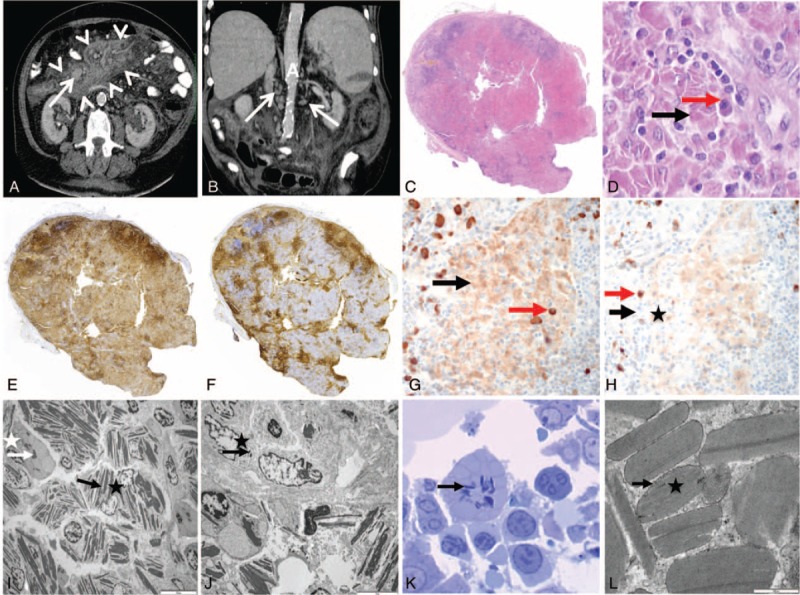
Computed tomography (CT) scan of the abdomen (A, B), lymph node (C–J), and bone marrow biopsy (K, L). (A) Axial view upon iode injection, showing mesenteric fat tissue hyperdensity (arrow heads) and mesenteric lymph node enlargement (arrow). (B) Coronal view upon iode injection showing multiple paraaortic lymph nodes (arrows). A = aorta. (C) General view of the lymph node (hematoxylin–eosin staining, original magnification ×25). Lymph node parenchyma is replaced with large sheets of pink cells corresponding to infiltrating macrophages. Few follicules persist on boundaries. (D) Pink area in lymph node (hematoxylin–eosin staining, original magnification ×400). Pink cells correspond to macrophages filled with crystals (black arrow). They are associated with some plasma cells (red arrow). (E) Immunoperoxidase anti-CD3 staining (original magnification ×25). (F) Immunoperoxidase anti-CD20 staining (×25). Normal T and B territories persist on boundaries of the lymph node and between the sheets of macrophages. (G) Immunoperoxidase anti-κ staining (×400). (H) Immunoperoxidase anti-λ staining (×400). There is a polyclonal staining pattern of plasma cells (red arrows), and crystals were predominantly stained with κ-antibody (black arrows). (I) Lymph node analysis on electron microscopy (×2000). Macrophage (black star) and some plama cell (white star) contain crystals (white and black arrows) in their cytoplasm. (J) Lymph node analysis on electron microscopy (×5000). Some crystals (black arrow) were found in endothelial cell (black star). (K) Bone marrow on electron microscopy (toluidine blue, ×1000). Some plasma cells contain crystals (arrow) in their cytoplasm. (L) Crystal analysis on electron microscopy (×50,000). Crystal (star) does not have periodicity and is lined by an endocytosis membrane (arrow).

A 69-year-old man was admitted to hospital in August 2013 for a very poor performance status, including a 15 kg weight loss in the last 6 months and bouts of fever. He had a history of myocardial infarction 17 years before, thromboembolic disease, and surgery for prostatic adenoma. The physical examination revealed small bilateral pleural effusions, several small peripheral lymph nodes, and moderate splenomegaly. Blood counts showed normochromic normocytic anemia with 68 g/L hemoglobin (normal range: 110–150 g/L), a lymphopenia (0.5 × 10^9^/L), and a normal platelet count. Laboratory analyses revealed an increased erythrocyte sedimentation rate (140 mm/h, normal <20 mm/h), elevated serum C-reactive protein (CRP, 137 mg/L, normal <6 mg/L), and increased serum β2-microglobulin (5.5 mg/L, normal <1.8 mg/L). The serum ferritin level was 445.7 μg/L (normal <219 μg/L). Serum calcium, Lactate deshydrogenase, serum IgG, IgA, and IgM levels were normal. Serum protein electrophoresis and immunofixation revealed an oligoclonal pattern (1 IgGκ, 1 IgGλ), with normal levels of polyclonal Igs. The serum free κ LC level was 293 mg/L (normal range: 1.7–3.7 mg/L), whereas the serum free λ LC was 34 mg/L (κ/λ ratio = 8.62). Renal function was normal (serum creatinine = 90 μmol/L; Modification of Diet in Renal Disease estimating Glomerular Filtration Rate = 75 mL/min/1.73 m^2^), but there was a moderate proteinuria (0.69 g/d), including free polyclonal Ig LC and 30% of a monoclonal κ LC. There was no biological evidence of a Fanconi syndrome. Peripheral immunophenotyping revealed a CD20+, CD5−, CD23+, CD10−, FMC7+, CD38− B cell monotypic population of κ type (Matutes score = 0). The blood karyotype was normal, and we did not detect a MYD88 mutation, thus making a diagnosis of Waldenstrom macroglobulinemia unlikely. Bone marrow aspirate included 1% plasma cells with a normal morphology and 15% normal lymphocytes. A monoclonal rearrangement of the immunoglobulin H locus was demonstrated by specific polymerase chain reaction. The erythroid lineage appeared normal on bone marrow smears, and the observed anemia likely related to systemic inflammation. Phenotypic analysis by flow cytometry revealed that 10% of bone marrow plasma cells were CD19−and CD56+. No LC restriction was noticed upon in situ hybridization studies. Searches for infections by HIV, Epstein Barr Virus, Cytomegalovirus, and Human Herpes Virus 6 viruses were all negative, as well as for aspergillosis, toxoplasmosis, and candidiasis. Tests for tuberculosis (intradermal tuberculin and Quantiferon) were negative. There was no lytic lesion on skeleton x-ray and the Positive Emission Tomography scan did not detect any hypermetabolic site. The computed tomography (CT) scan revealed upper and subdiaphragmatic lymphadenopathy with a major mesenteric involvement resembling peritoneal carcinomatosis, along with moderate splenomegaly (17.5 cm; Figure 1A and B). A diagnostic inguinal lymph node excision biopsy was undertaken: 2 lymph nodes of 2 cm each were studied and both presented with numerous macrophages filled with crystals. In addition, a mesenteric biopsy showed similar aspects in the abdominal adipose tissue.

The above findings led to a diagnosis of CSH with multiple lymph node involvement and kidney disease. Persistent urinary excretion of a significant amount of a monoclonal LC strongly suggested that a plasma cell dyscrasia was the cause of the observed pathology. A combination therapy, including bortezomib and dexamethasone, was initiated in November 2013. Each cycle of the regimen consisted of subcutaneous bortezomib (1.3 mg/m^2^) on days 1, 4, 8, and 11; dexamethasone (40 mg) on days 1, 4, 8, 11 of a 21-day cycle. After 4 cycles of chemotherapy, a dramatic improvement of the general status was observed, with serum κ LC level falling to 39 mg/L (κ/λ ratio = 2.16) and a normal sized spleen. The panniculitis decreased by 60% after treatment on CT scan. The serum CRP level also returned to normal. The treatment was stopped without consolidation or maintenance. The patient showed persistent clinical and biological improvement after a 12-month follow-up.

## MATERIALS AND METHODS

A signed informed consent was given by the patient. Approval by an ethic committee was not required because all studies were included in a standard clinical management.

Formalin-fixed paraffin-embedded tissues were cut into 3-μm thick sections and stained with hematoxylin and eosin or used for immunohistochemical (IHC) studies. IHC analyses were performed with the Bond-Max automated immunostainer (Leica Microsystems, Wetzlar, Germany) using the following antibodies: anti-CD3, -CD20, -CD68, -κ and -λ LCs (Novocastra, Newcastle upon Tyne, UK).

Complementary DNA was obtained by reverse transcription of RNA extracted from a bone marrow aspirate, using High Capacity cDNA Archive Kit (Applied Biosystems, Carlsbad, CA). Three distinct PCR amplifications were performed using a 3′ primer complementary to the κ constant region and 3 different 5′ primers representing consensus sequences of leader regions of Vκ subgroups, as previously described.^6^ PCR products were cloned into pCR2.1 TOPO vector (Invitrogen, Carlsbad, CA), and DNA sequencing was performed using Big-Dye terminators (Applied Biosystems) on a 16-capillaries electrophoresis system 3130 XL (Applied Biosystems). Sequences were analyzed using FinchTV software and aligned on Multalin (http://multalin.toulouse.inra.fr/multalin/).

## RESULTS: PATHOLOGIC FEATURES AND MONOCLONAL LC SEQUENCE

The lymph node was diffusely infiltrated with large sheets of macrophages filled with crystalline needle-like inclusions (Figure 1C and D). The residual lymph node parenchyma was made of very rare follicles (CD20+) and few T-cell zones (CD3+). Rare polytypic plasma cells were dispersed within sheets of macrophages (Figure 1E and F). The crystal inclusions stained mostly with anti-κ antibody (Figure 1G and H). On electron microscopy, crystals were found mainly within macrophages, but also inside a few plasma cells (Figure 1I) and some endothelial cells (Figure 1J).

The bone marrow biopsy appeared normal, except for few crystal-storing macrophages that were detected by electron microscopy (Figure 1K and L).

**FIGURE 2:**
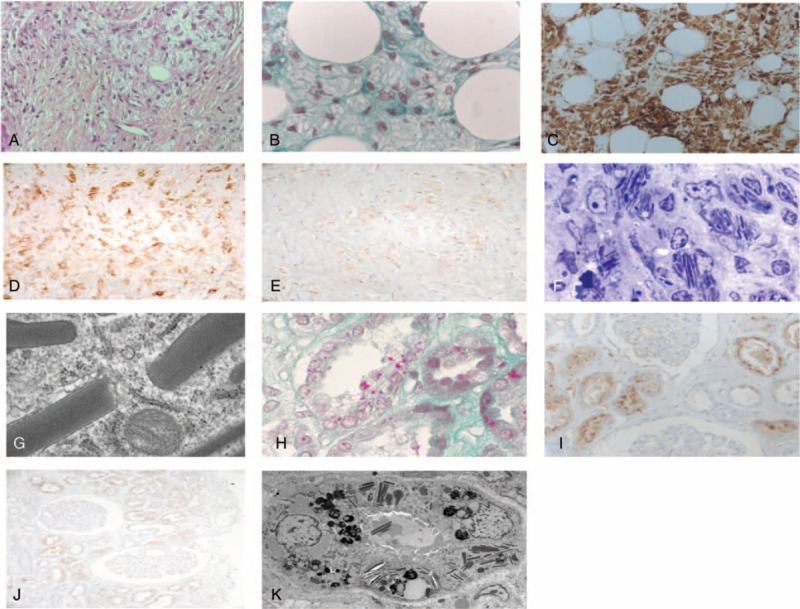
Kidney biopsy. (A, B) Numerous pseudo-Gaucher macrophages containing crystalline inclusions, located in a prominent perirenal fibrous tissue (A, hematoxylin–eosin–saffron staining, original magnification ×400) and surrounding perirenal adipocytes (B, Masson trichrome, original magnification ×1000). (C–E) Immunohistochemical staining of the crystal-containing macrophages with anti-CD68 (C, original magnification ×400), anti-κ LC (D, ×400), and anti-λ LC (E, ×400) antibodies. (F, G) Intramacrophagic needle-like crystals. Semithin section (F, toluidin blue staining, original magnification ×400) and electron microscopy (G). (H) Fuchsinophilic crystals within cytoplasms of tubular epithelial cells (Masson trichrome staining, original magnification ×1000). (I, J) Immunohistochemical staining of tubular crystals with anti-κ antibody (I, ×400) and anti-λ antibody (J, ×400). (K) Needle-like crystals within cytoplasms of tubular epithelial cells (electron microscopy). LC = light chain.

The kidney biopsy showed striking fibrosis of the perirenal adipose tissue, which was infiltrated with numerous large macrophages loaded with crystalline material mimicking Gaucher cells (Figure 2A and B). Few similar cells were also present in the renal interstitium. These cells were CD68-positive macrophages (Figure 2C), and inclusions predominantly stained with anti-κ antibody (Figure 2D and E). Macrophage crystals were also detected in the cytoplasm after toluidine blue staining of semithin sections (Figure 2F). Congo red staining was negative. Electron microscopy revealed needle-like crystals within the cytoplasm of macrophages (Figure 2G). Similar κ-positive needle-like inclusions were also detected in the cytoplasm of kidney tubule epithelial cells (Figure 2H–K).

**FIGURE 3:**
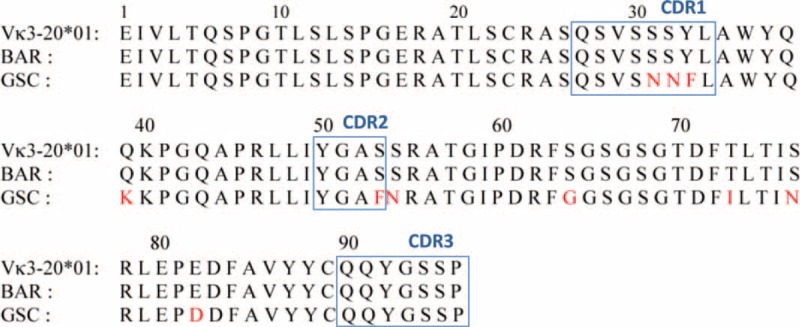
Amino acid sequence of the κ LC. Amino acid sequence of the patient's monoclonal κ LC V region (Bar), aligned with the translated germ line Vk3-20^∗^01 and one previously published case (GSC) of CSH.^6^ Mutations are indicated in red. Boxes correspond to CDR1, CDR2, and CDR3 regions. CDR = complementarity determining regions, CSH = crystal-storing histiocytosis, LC = light chain.

Six out of 10 Vκ3 subgroup sequences were identical and corresponded to a κ LC encoded by the germline Vκ3-20^∗^01 gene segment rearranged with Jκ2^∗^01 (International ImMunoGenetics information system IMGT, http://www.imgt.org/). Surprisingly, both V and J domains were 100% identical to the corresponding germline segments (Figure 3). To our knowledge, this is the first reported case of a CSH monoclonal LC without somatic mutation.

## DISCUSSION

CSH may occur not only in several etiological contexts including most frequently monoclonal gammopathy, but also in certain infectious diseases such as tuberculosis, or autoimmune conditions such as rheumatoid arthritis.^2^ The latter diagnoses were ruled out. In the present case, although an underlying hematological disease could not be precisely defined, a monoclonal κ LC was clearly and continuously excreted in the urine. There was no evidence of an overt lymphoproliferative disease based on lymph node, bone marrow, and kidney biopsies.

Few biochemical analyses of pathogenic LC in CSH have been performed so far.^7^ In the present case, evidence of a lymphoproliferative disease relied on the excretion of a urinary monoclonal κ LC and bone marrow infiltration by a small B-cell clone, as revealed by molecular analyses of mRNA.

Mesenteric involvement by CSH may erroneously suggest a diagnosis of peritoneal carcinomatosis at CT scan examination. Besides typical crystal-containing macrophages, the kidney biopsy revealed a striking fibrous reaction in perirenal adipose tissue. Such features are reminiscent of a previously reported case of CSH mimicking “multifocal fibrosclerosis” with notable mesenteric and peritoneal fibrosis.^8^ Although CSH frequently involve bone marrow, kidneys, thyroid, cornea, and skin, it can also manifest with peritoneal and retroperitoneal localizations associated with prominent fibrous reaction.

CSH patients with kidney involvement most usually present with acute or chronic renal failure and metabolic markers of Fanconi syndrome.^9–12^ By contrast, our patient presented only with a moderate proteinuria of tubular origin together with a prominent monoclonal κ LC, but no other features of tubular dysfunction and a normal serum creatinine level. On the contrary, renal lesions were obvious and diffuse, with crystal formations in both tubular epithelial cells and macrophages.

The disease seems clearly related to κ-type LC, suggesting that intrinsic factors of these LC might cause their accumulation inside phagosomes of macrophages, where they seem to resist proteolysis and form crystals. A similar mechanism has been well documented in cases of Fanconi syndrome,^13,14^ a condition that is not rarely associated with CSH.^6,15^

Drawing hypotheses on the mechanisms of macrophage activation and crystal storing in CSH is prevented by the scarcity of molecular data. In 1 case, the LC variable region derived from the Vκ1-33 gene segment and was shown to display unusual amino acid substitutions.^5^ The authors proposed that a conformational alteration was a probable crucial factor in the pathogenesis of CSH. In a more recent study of 3 CSH cases, El Hamel et al^6^ found distinctive structural features of the κ LC variable regions, as compared with known Fanconi syndrome LC sequences. They proposed that specific somatic mutations of the V regions would determine their propensity to induce either CSH or Fanconi syndrome, or both.

It is quite striking that in our case, there was not a single mutation of the κ LC: the sequence was strictly identical to the corresponding germline gene segments. Of note, the LC V region from our patient is encoded by a rearranged gene segment, Vκ3-20^∗^01/Jκ2^∗^01, that had also been identified in a previous case.^6^ However, the latter displayed 10 somatic mutations, whereas the remarkable absence of somatic mutation in our patient's LC suggests that the Vκ3-20 germline sequence by itself presents with a pathogenic potential for CSH. Larger series of observations including molecular analyses are required for confirming this hypothesis.

In the absence of an identified lymphoproliferative disease, a combination of bortezomib plus steroid was given to the patient because it is currently one of the best treatment for plasma cell disorders. Indeed, the patient's general status improved dramatically, and biological and pathological features almost disappeared. However, the serum κ:λ ratio remained slightly elevated (2.16), and the mesenteric lymph node involvement remained detectable.

Another reported case of MGUS-related CSH was successfully treated with the combination of bortezomib, thalidomide, and dexamethasone.^16^ Finally, we also recently reported on a CSH case with ocular involvement and Fanconi syndrome associated with MGUS, who dramatically responded to several courses of bortezomib followed by autologous stem cell graft.^17^

Future molecular and pathophysiological studies of new CSH cases will hopefully improve our understanding of this condition, which would then allow designing focused therapies that would target the pathogenic properties of the responsible LC.
